# 566. Development of a Prototype Carbapenem-Resistant Acinetobacter baumannii-calcoaceticus complex (CRABC) Assay for use on the cobas® 5800/6800/8800 System Utility Channel

**DOI:** 10.1093/ofid/ofad500.635

**Published:** 2023-11-27

**Authors:** Natacha Martins Sorenson, Hai Nguyen, Arrash Moghaddasi, Aishwarya Sathish, Indira Somanathan, Letong Jia, Patrick Lin, Xun Zhuang, Colin Lam, Brian Lee, Kyle C Cady

**Affiliations:** Roche Diagnostics, Santa Clara, California; Roche Diagnostics, Santa Clara, California; Roche Diagnostics, Santa Clara, California; Roche Diagnostics, Santa Clara, California; Roche Diagnostics, Santa Clara, California; Roche Diagnostics, Santa Clara, California; Roche Diagnostics, Santa Clara, California; Roche Diagnostics, Santa Clara, California; Roche Diagnostics, Santa Clara, California; Roche Diagnostics, Santa Clara, California; Roche Diagnostics, Santa Clara, California

## Abstract

**Background:**

Carbapenem-resistant *Acinetobacter baumannii-calcoaceticus* complex (CRABC) is recognized as an urgent/critical antimicrobial resistant threat by the Centers for Disease Control and Prevention and World Health Organization. The development of new antibiotics targeting CRABC is an urgent unmet medical need that Roche is tackling through development of a novel antimicrobial compound (zosurabalpin). A significant challenge for the planned pathogen-focused clinical trial is the need to rapidly identify eligible patients with CRABC infections. Here, we describe a prototype assay for the rapid detection of CRABC causing HAP/VAP or bloodstream infections (BSI).

**Methods:**

This prototype assay was developed for the qualitative detection of *A. baumannii- calcoaceticus* complex (ABC) organisms harboring the most prevalent associated carbapenemases genes (*bla*OXA-23, *bla*OXA-24, *bla*OXA-58, and *bla*NDM). This work entailed the assay design and optimization for use on the automated, high-throughput (HTP) **cobas®** 5800/6800/8800 Systems, including primers/probes, sample processing, and result interpretation for three sample types (BAL, sputum, and positive blood culture). The assay utilizes the commercially available **cobas omni** Utility Channel Kit, a defined oligo pool, controls, and an off-instrument analysis package. Assay inclusivity, exclusivity, limit of detection (LoD), and specificity were evaluated using archived or contrived clinical specimens.

**Results:**

The CRABC prototype assay had 100% inclusivity for ABC and claimed resistance markers and 100% exclusivity for other bacteria commonly found in respiratory or positive blood culture samples based on *in silico* and wet lab testing. Using preliminary interpretive cutoffs, high specificity (100%) was observed with negative clinical samples (n=90, per sample type) and LoDs of 5280 (95% CI: 4010-9166), 6180 (95% CI: 4548-11285) and 1250 CFU/mL (95% CI: 1050-1750) were observed for BAL, sputum and positive blood culture, respectively.

**Conclusion:**

This CRABC prototype assay for use on the automated, HTP **cobas®** 5800/6800/8800 Systems could be adopted to rapidly identify patients with CRABC-associated HAP/VAP or BSI for enrollment in a pathogen-focused clinical trial.

**Disclosures:**

**Natacha Martins Sorenson, PhD**, Roche: Grant/Research Support **Hai Nguyen, n/a**, Roche: Grant/Research Support **Arrash Moghaddasi, n/a**, Roche: Grant/Research Support **Aishwarya Sathish, n/a**, Roche: Grant/Research Support **Indira Somanathan, n/a**, Roche: Grant/Research Support **Letong Jia, n/a**, Roche: Grant/Research Support **Patrick Lin, n/a**, Roche: Grant/Research Support **Xun Zhuang, PhD**, Roche: Grant/Research Support **Colin Lam, n/a**, Roche: Grant/Research Support **Brian Lee, MD**, GE Healthcare: Stocks/Bonds|Invitae: Stocks/Bonds|Merck: Stocks/Bonds|OPKO Health: Stocks/Bonds|Roche Diagnostics: Employment|Roche Diagnostics: Stocks/Bonds|Spero Therapeutics: Stocks/Bonds **Kyle C. Cady, PhD**, Roche: Grant/Research Support|Roche: Stocks/Bonds

